# Chikungunya Virus Infections in Military Deployments in Tropical Settings—A Narrative Minireview

**DOI:** 10.3390/v11060550

**Published:** 2019-06-14

**Authors:** Hagen Frickmann, Ottmar Herchenröder

**Affiliations:** 1 Department of Microbiology and Hospital Hygiene, Bundeswehr Hospital Hamburg, 22049 Hamburg, Germany; frickmann@bnitm.de; 2 Institute for Medical Microbiology, Virology and Hygiene, University Medicine Rostock, 18057 Rostock, Germany; 3 Institute for Experimental Gene Therapy and Cancer Research, Rostock University Medical Center, 18057 Rostock, Germany

**Keywords:** chikungunya virus, military deployment, epidemiology, soldiers, risk assessment

## Abstract

Chikungunya fever is a vector-borne viral disease in subtropical and tropical areas of endemicity. Apart from the burden on local populations, chikungunya virus infection also poses a risk for travelers and, in particular, soldiers during prolonged deployment-associated outdoor activities. The absence of rapid diagnostic tests makes surveillance challenging during military deployments in war and crisis zones with restricted medical infrastructure. Consequently, both historical and up-to-date surveillance data from battlefields are scarce. From several studies and postdeployment assessments, some information on the epidemiology of chikungunya virus infections in deployed military personnel is nevertheless available. The few published data homogeneously suggest a low infection risk in the endemic setting. During outbreaks, however, the infection risk of military personnel is comparable to that of the local population. Infection clusters of soldiers without pronounced outdoor activity have been reported under such circumstances as well. In spite of efforts focusing on the development of a chikungunya virus vaccine, no licensed product is available so far.

## 1. Introduction

**Figure 1:**
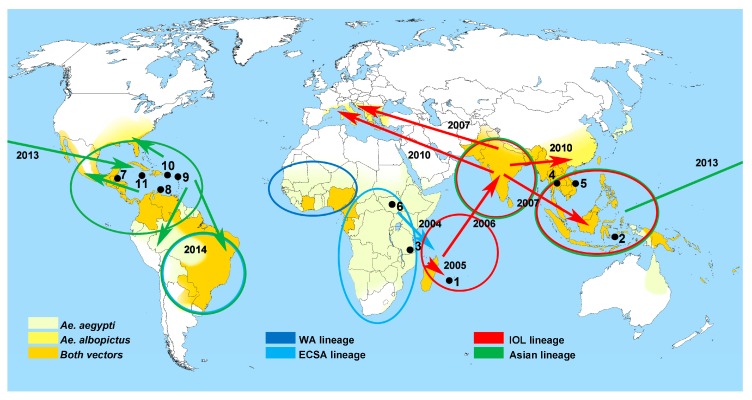
Countries or regions with chikungunya virus (CHIKV) endemicity and/or outbreaks mentioned in this review in the order of appearance (black dots and numerals 1–11). 1. Réunion; 2. Malay Archipelago; 3. Makonde/Tanzania; 4. Thailand; 5. Vietnam; 6. Southern Sudan; 7. Belize; 8. Curaçao; 9. Saint Martin; 10. Puerto Rico; 11. Jamaica. Arrows with year dates indicate global spread of CHIKV as described by Young, 2018 [7]. Map colorings show infestations of countries and areas with relevant arthropod vectors according to Rezza and Weaver, 2019 [8]. Information on CHIKV lineages were adapted from Weaver and Lecuit, 2015 [4]. Map template source: Petr Dlouhý, Wikimedia Commons.

Chikungunya virus (CHIKV) is a single-stranded RNA virus of the genus *Alphavirus* in the family *Togaviridae* [1]. CHIKV infections are predominantly transmitted by the daytime-active vectors *Aedes aegypti* and *Aedes albopictus* in subtropical and tropical settings [1]. Over the last couple of years, CHIKV ran rampant along the shores of the Indian Ocean, causing considerable outbreaks in several tropical African countries and islands including La Réunion Island and the Malay Archipelago [2,3]. Subsequently, the virus managed to cross the Pacific Ocean to enter the Americas by gaining initial footholds in the Caribbean basin [4]. The major cause of the rapid spread of the virus around the globe within only a few years was a single mutation in its envelope which increased viral fitness in *Aedes albopictus* [5]. Considering this adaption of CHIKV to another vector that bears the potential of spreading alongside a probable climate change to more temperate areas of the world in decades to come [6], one may be prepared to see outbreaks of chikungunya fever epidemics further north and south than along its current endemicity range (Figure 1). Taken together, besides Zika virus, CHIKV is another “arbovirus on the move” as recently described in detail by Paul Young, 2018 [7] and Rezza and Weaver, 2019 [8]. Similar to Zika virus, however, increased numbers of CHIKV infections are often associated with temporary outbreak events [9], leading to temporally increased infection risks in contrast to arbovirus infections such as dengue fever, with continuously increasing case numbers [7,8].

Accordingly, apart from the impact on local populations as well as on travelers, CHIKV also poses a threat to soldiers or peace-keeping forces who are deployed in areas of endemicity and exposed due to their professional outdoor activity. Up-to-date maps of areas of autochthonous CHIKV transmission can be monitored at the Centers of Disease Control and Prevention [9]. Chikungunya fever disease is associated with elevated body temperature, rash, and severe, often long-lasting polyarthralgia [10,11] with considerable impact on soldiers’ health and ability to fulfil their duty on deployment. Persistent synovitis as described for a US soldier deployed to Central America may occur [12]. In line with the symptoms, the term “chikungunya” is derived from the term “kungunyala” of the Makonde language, meaning “to become contorted” [1,13]. The early symptoms of chikungunya fever are clinically indistinguishable from other febrile tropical virus infections such as dengue fever [11]. This circumstance makes differential diagnosis rather challenging, since on-site surveillance in the field on deployment requires sophisticated mobile diagnostic equipment or high-level host nation support.

Certainly, CHIKV is not the only viral agent with potentially harmful effects on the health of deployed soldiers. Basically, deployed soldiers are exposed to the same infection risks as civilian travelers or deployed civilian health care workers, with transmission routes ranging from smear infections, like in the case of viral gastroenteritis, to viral infections transmitted by stings, bites, or droplets from arthropods or other animals or human-to-human infections including sexually transmitted viral diseases [14,15]. Only a few viral infection risks associated with traveling or deployments are vaccine preventable, such as the arthropod-borne yellow fever and Japan B encephalitis, tick-borne encephalitis, as well as rabies or hepatitis A and B infections [16]. 

This narrative minireview concentrates on and summarizes experiences of international military medical services with the epidemiology of chikungunya infections in deployed personnel and preventive approaches for the deployment situation. The literature review was based upon PubMed/Medline searches (www.pubmed.org, last accessed May 2019), including search terms such as “chikungunya”, “soldier”, and “military” as well as others in varying compositions.

## 2. Early Experience of Deployed Soldiers in Southeast Asia in the 20th Century

While dengue fever was a previously known menace for deployed soldiers in tropical settings during World War II, with around 90,000 infected US soldiers [17], CHIKV could not be detected at that time. First described in 1952 during an outbreak in the African Makonde region, nowadays a part of Tanzania [1], chikungunya infections as relevant problems for deployed soldiers were first described by US forces in Southeast Asia. Between 1962 and 1964, 1% of American residents in Bangkok, Thailand, were infected either with dengue or chikungunya virus. While American military personnel were particularly affected by a dengue outbreak in Ubol, Eastern Thailand during this time, individual CHIKV infections among Americans could be confirmed in the course of the US Medical Unit Study, and the majority of dengue-or-chikungunya-like illnesses remained diagnostically unresolved (Table 1) [18].

During the Vietnam War, several cases of CHIKV infections were confirmed and studies focusing on fever of unknown origin (FUO) during this long-lasting conflict estimated that up to 15% of FUO cases might have either been caused by dengue or chikungunya fever [19,20,21]. However, due to the lack of sophisticated on-site diagnostic options, the precise distribution patterns of CHIKV infections during the Vietnam War remains speculative [22]. While Deller and Russell [19] reported 10 cases with confirmed chikungunya infections among 110 FUO cases (9%), other studies described only 1 out of 94 cases or even 0 out of 688 cases [20,22]. Accordingly, the real case rates during the Vietnam War due to CHIKV remain uncertain but have presumably been small.

## 3. Low Infection Rates in the Endemic Setting

Since the threat of vector-borne transmission of infectious diseases is imminent for deployed soldiers on outdoor activities in endemic areas, the observed burden of infections in the endemic situation seems to be low [12] by means of objective numbers of identified military patients with proven CHIKV infections. Although systematically collected screening data for military populations are not available, this conclusion can be drawn from the results of several cross-sectional studies assessing soldiers deployed to areas where CHIKV is endemic.

Between fall of 2012 and spring of 2013, 632 paired serum samples of Mongolian peacekeepers deployed to Southern Sudan had been assessed for potential seroconversion against CHIKV after they had suffered from febrile illnesses at the deployment site. Although this country borders the area of known CHIKV endemicity with a low prevalence of <2% even in fever patients [28], not even a single seroconversion suggested infections with this virus, while immunologically relevant contacts with other pathogens such as rickettsiae, West Nile virus, *Coxiella burnettii*, dengue virus, and *Leptospira* spp. were infrequently described [19].

A cohort of 124 Dutch soldiers deployed for a median of eight weeks to Belize, Curaçao, or Saint Martin in 2017 was assessed at least 14 days after returning for signs of arthropod-borne viral infections that may have been acquired on deployment. A total of 19 servicemen were tested serologically due to a medical history compatible with acute CHIKV infection. IgG antibodies against the virus were only detected in 1 out of these 19 soldiers. The seropositive serviceman had been deployed to Saint Martin [20]. During the outbreak in 2013 and 2014, Saint Martin had reported an attack rate of 1.76% [29].

German soldiers deployed abroad are invited to take part in voluntary returnee screenings at the Department of Infectious Diseases and Tropical Medicine at the Bernhard Nocht Institute in Hamburg, Germany. Generally, this service is widely accepted by soldiers in order to exclude infections or infestations after tropical deployments with increased infection risks, for example, UN observers on duty in locations without food and accommodation provided such as in standardized field camps [30,31]. Generally, these soldiers are more exposed to local risk factors as they share housing and nutritional conditions with the local population. Within the previous 10 years with an average of about 200 assessed returnees per year from various deployment sites comprising Afghanistan, Argentina, Bosnia and Herzegovina, Brazil, the Central African Republic, China, the Democratic Republic of the Congo, Djibouti, Ethiopia, French Guyana, Gabon, Ghana, Indonesia, Jamaica, Kosovo, Lebanon, Liberia, Mali, Malta, Morocco, Nigeria, Pakistan, Panama, Senegal, Somalia, South Sudan, Sudan, Tanzania, Thailand, Uganda, Uzbekistan, Venezuela, Vietnam, Zimbabwe and unknown or multiple deployment settings, not a single case of CHIKV infection has been observed in 2153 soldiers.

## 4. Outbreaks Affecting Military Personnel

In contrast to the apparently low frequency of CHIKV infections in soldiers in the endemic situation, higher attack rates have to be considered in outbreak settings. During the well-documented CHIKV outbreak in Réunion during 2005 and 2006, French forces deployed on this island were among the 770,000 inhabitants from which 35% were infected during the initial six months of the outbreak. Screening data from a military cohort suggested that the attack rate within the soldiers was similar to the one in the local population [21,25]. Detailed epidemiological data are available from a cohort of 662 French military policemen who were deployed in Réunion Island during the outbreak and responded to a questionnaire-based assessment. This cohort mainly consisted of young or middle-aged men with a median age of 40 years. While 23.9% self-reported symptoms compatible with CHIKV infections, the total seroprevalence (i.e., proof of specific IgM antibodies or IgG antibodies or both) was 19.3%, with only 3.2% asymptomatic cases. Chronic disease with pains in joints, bones, or both was reported by 93.7% of symptomatic patients with considerable impact on their duty. Acute fever was documented for 86.5% of these soldiers, a rash in 54.4%, and swollen articulations in 44.8% of the cases. Signs of hemorrhage were infrequently observed, with 4.1% hematoma and 2.6% minor bleedings. All infected soldiers reported tiredness, a condition that at least partially affected their duties [25].

More than this, even 30 months after CHIKV infection in Réunion Island, some infected French military policemen still reported considerable rheumatic symptoms and fatigue. A subset of 37.4% of them, who considered themselves as “nonhealed”, reported ongoing substantial limitations in their activities, with all of them suffering from pain [32]. Unfortunately, both assessments of the affected French military policemen did not provide any details on potential risk factors for severe or prolonged clinical courses [25,32]. In a retrospective assessment, French epidemiologists tried to identify risk factors for long-persisting disease over about 30 months in a multiple correspondence analysis based on the data from the infected military policemen from Réunion Island. Sick leave with a duration of more than four days, joint swelling, depressed mood, as well as early chronic arthritis were associated with long-term-persisting arthritis and self-perceived nonrecovery [33].

Between 2010 and 2016, the US military recorded 78 confirmed cases of chikungunya fever among their active and reserve service members. Of those 78 cases, 64 were reported during the peak season in 2014 [27] associated with the establishment of CHIKV in the Western Hemisphere in December 2013 [34]. The highest proportion (*n* = 50) of infections was acquired in Puerto Rico. Of note, only 50% of the 78 chikungunya patients had records of confirmed positive laboratory results in the US Defense Medical Surveillance System [27].

In detail, a case series of five CHIKV-infected US soldiers at the US Forward Operating Location, 429th Expeditionary Operations Squadron based in Curaçao, had been reported between November 2014 and January 2015. So far, this had been the largest single cluster of chikungunya cases in the US Air Force. All patients were male. The infected individuals experienced relatively mild symptoms with none of the patients requiring hospitalization or repatriation. Good responses to pharmaceutical pain management were reported. Interestingly, the majority of the duty day of the affected soldiers was neither outdoors nor at a mosquito-prone location [26].

In an earlier publication, even 118 cases of active US service members with CHIKV infections had been reported between January 2014 and February 2015. The main sites of infections were Puerto Rico (*n* = 85), Jamaica (*n* = 7), and Curaçao (with the five patients described above) next to regions with single cases [22,26]. With the decline in civilian cases in the Western Hemisphere after the peak of chikungunya virus infections in 2014, the number of affected US soldiers deployed in Central America declined substantially [27].

To date, a handful of genetically distinct CHIKV lineages has been described as reviewed by Zeller et al. [35]. Accepted lineages are the West African lineage (WA); the East, Central, and South African (ECSA) lineage; the Asian lineage; and the Indian Ocean (sub)lineage (IOL) [36]. With this background and the above-stated enhanced transmissibility of a rather recent CHIKV strain by *A. albopictus*, it would be interesting to know whether or not one or another lineage or strain has had a stronger impact on personnel in field settings. None of the above reports, however, allows respective unambiguous statements. During the Réunion outbreak in 2005 and 2006, a higher disease burden and mortality as well as severe mother-to-child transmissions were reported [37,38]. Although a large number of deployed individuals was also affected during this outbreak and with a certain percentage of them having reported severe and lasting illness, it remains too speculative to determine whether IOL may have a higher pathogenic potential than others lineages.

## 5. Diagnostic and Preventive Strategies

The diagnosis of chikungunya fever on military deployments is challenging due to the absence of rapid and reliable diagnostic test systems within field settings [11]. Especially in resource-limited tropical areas such as Sub-Saharan Africa, to our knowledge, only a few centers such as the CDC Uganda Viral Research Institute (UVRI) in Entebbe can provide definitive testing based on PCR from patients’ blood, as it is organized for US troops deployed there on behalf of the SOCAFRICA Surgeon’s Office [11]. Other locations where reliable chikungunya testing can be provided for deployed troops or travelers are listed by the Pan American Health Association [1,39]. PCR in combination with IgM testing is considered reliable during the first eight days of illness, while the immunoglobulin class switch can be expected 10–14 days after the onset of clinical symptoms. Virus isolation is restricted to reference laboratories and is usually unavailable at military deployment sites [1]. When treating chikungunya patients, one has to bear in mind for epidemiological and hygienic reasons that viremia can lead to virus transmission by feeding mosquitos for up to one week [1]. In daily practice, however, the US military medical service did not observe this phenomenon during the management of chikungunya fever cases during the outbreak in the Western Hemisphere in 2014 [22].

Neither specific antiviral treatment nor a licensed vaccine for human chikungunya virus infections are available, stressing the importance of transmission prevention by using permethrin-treated uniforms, repellents for unprotected skin, and bed-nets during periods of rest [1,11,40]. Options for permethrin treatment, however, depend on the type of uniform used, and the effectiveness of repellent strongly depends on individual compliance [35]. The few available studies on CHIKV infections in soldiers were, however, neither designed nor powered to answer the question of the effectiveness of such traditional preventive approaches on actual CHIKV transmission prevention in deployed soldiers. Further, no international standardization of exposure prevention against arbovirus infections of soldiers on deployment has ever been enforced. Respective studies and coordinated action are nevertheless desirable.

Efforts are ongoing towards the development of a vaccine against CHIKV. Decades ago, the attenuated chikungunya live vaccine TSI-GSD-218 was developed by US military investigators at the Salk Institute—Government Services Division. However, production was stopped in 1994 and the development program was terminated in 1998 [41]. Associated with the increased spread of chikungunya virus since the middle of the last decade, TSI-GSD-218-related information was provided to civilian developers and producers. Nevertheless, a licensed vaccine is not available so far [42]. A recent phase II trial for a vaccine against CHIKV performed by Austrian and German scientists raises new hope to combat this pathogen. These researchers used a measles-virus-vectored chikungunya virus vaccine that yielded a strong neutralizing antibody response and no adverse events related to the vaccination [43]. In addition, it was shown that this vaccine protects nonhuman primates from viremia, disease, and proinflammatory blood parameters upon challenge after vaccination [44].

## 6. Conclusions

In summary, chikungunya virus infections are realistic threats to deployed soldiers in endemic areas due to the vector-borne mode of transmission. Apart from outbreak settings, however, the frequency of transmission in deployed soldiers in the tropics seems to be low [23,24]. Comparisons with other arbovirus infections are problematic. Similar to CHIKV infections in soldiers, systematic surveillance in international military forces apart from point-prevalence studies and national registers is also widely missing for other arbovirus infections. Therefore, it is difficult to draw evidence-supported conclusions. During outbreaks, however, CHIKV infection rates increase similarly to those observed in the local population [21,25]. Infection clusters even in servicemen with comparably low risk of contact with vectors have been described [26]. Due to the lack of licensed vaccines and virus-specific treatment, permethrin-treated uniforms as well as repellent and bed-net use remain the methods of choice to prevent CHIKV transmission to soldiers and peacekeepers on deployment in endemic settings [1,11,40].

## Figures and Tables

**Table 1:** Reported chikungunya virus infections in soldiers and service members working for the military.

Military Servicemen	Region	Setting	Confirmed Cases	References
**Experience from areas of endemicity**				
US service members	Thailand	Endemic, 1962–1964	<1%, few confirmed cases in 627 Americans	[18]
US service members	Vietnam	Endemic, Vietnam War	0–9% (0/688 [22], 1/94 [19], highest: 10/110 patients with febrile disease according to [20])	[19,20,22]
Mongolian armed forces	Southern Sudan	Endemic, 2012–2013	0% (0/632)	[23]
Dutch armed forces	Belize, Curaçao, Saint Martin	Endemic, 2017	1% (one case, Saint Martin, 1/124)	[24]
German armed forces	Multiple tropical deployment settings	Endemic, 2006–2019	0% (0/2,153)	Own data
**Experience from outbreak scenarios**				
French military policemen	Réunion	Epidemic, 2005–2006	19.3% (128/622)	[25]
US service members	Western Hemisphere, special focus on Puerto Rico, Jamaica, Curaçao	Epidemic, 2014–2015	118 confirmed cases, no denominator listed	[22,26]
US military personnel only	Western Hemisphere, special focus on Puerto Rico	Epidemic, 2010–2016	78 confirmed cases, no denominator listed	[27]
